# Mapping Challenging Mutations by Whole-Genome Sequencing

**DOI:** 10.1534/g3.116.028316

**Published:** 2016-03-04

**Authors:** Harold E. Smith, Amy S. Fabritius, Aimee Jaramillo-Lambert, Andy Golden

**Affiliations:** National Institute of Diabetes and Digestive and Kidney Diseases, National Institutes of Health, Bethesda, Maryland 20892

**Keywords:** forward genetics, variant detection, complex alleles, SNP mapping

## Abstract

Whole-genome sequencing provides a rapid and powerful method for identifying mutations on a global scale, and has spurred a renewed enthusiasm for classical genetic screens in model organisms. The most commonly characterized category of mutation consists of monogenic, recessive traits, due to their genetic tractability. Therefore, most of the mapping methods for mutation identification by whole-genome sequencing are directed toward alleles that fulfill those criteria (*i.e.*, single-gene, homozygous variants). However, such approaches are not entirely suitable for the characterization of a variety of more challenging mutations, such as dominant and semidominant alleles or multigenic traits. Therefore, we have developed strategies for the identification of those classes of mutations, using polymorphism mapping in *Caenorhabditis elegans* as our model for validation. We also report an alternative approach for mutation identification from traditional recombinant crosses, and a solution to the technical challenge of sequencing sterile or terminally arrested strains where population size is limiting. The methods described herein extend the applicability of whole-genome sequencing to a broader spectrum of mutations, including classes that are difficult to map by traditional means.

The advent of next-generation sequencing technology has transformed our ability to identify the genetic variation that underlies phenotypic diversity. It is now feasible to perform whole-genome sequencing (WGS) in a matter of days and at relatively low cost. By comparing the data to an available reference genome, one can determine the complete constellation of sequence variants that are present in the sample of interest.

WGS has proven a boon to experimental geneticists who utilize classical, or forward, genetics (*i.e.*, random mutagenesis and phenotypic screening) in model organisms. Although those techniques have been employed for more than a century (Morgan, 1911a, 1911b), determination of the mutation responsible for the observed phenotype remains the rate-limiting step in most cases. Mutation identification by WGS compares favorably to traditional techniques, such as linkage mapping and positional cloning, in terms of speed, labor, and expense (Hobert 2010; Bowerman 2011; Hu 2014). Consequently, the technology has been adopted in a wide variety of model species, including *Caenorhabditis elegans* (Sarin *et al.* 2008), *Drosophila melanogaster* (Blumenstiel *et al.* 2009), *Escherichia coli* (Barrick and Lenski 2009), *Schizosaccharomyces pombe* (Irvine *et al.* 2009), *Arabidopsis thaliana* (Laitinen *et al*. 2010), *Saccharomyces cerevisiae* (Birkeland *et al.* 2010), *Dictyostelium discoideum* (Saxer *et al*. 2012), *Chlamydomonas reinhardtii* (Dutcher *et al*. 2012), and *Danio rerio* (Bowen *et al*. 2012).

A great advantage of forward genetic screening is the freedom from constraint on the types of alleles recovered; the only requirement is that the mutation produces the phenotype of interest. That property stands in contrast to techniques for reverse genetic screening, such as RNA interference (Fire *et al.* 1998), in which the molecular target is known but the ability to modify its activity is limited to a reduction of gene function. Although that category of mutation (reduction or loss of function) represents the class most commonly recovered in forward genetic screens, it is also possible to generate alleles that increase the level or activity of the gene product, reverse normal gene function, or produce novel activity. However, the expanded repertoire of variants accessible by random mutagenesis is accompanied by an increasing complexity in analysis. Although forward genetic screens are typically designed to obtain recessive alleles, they can also recover dominant, semidominant, and even multigenic mutations. The latter categories of alleles are challenging to map by traditional methods, such as linkage to morphological or molecular markers, due to the complexities of zygosity (for dominant and semidominant alleles) or the dependence of the phenotype upon independently segregating loci (for multigenic traits). As a practical consequence, preference is given to recessive, monogenic alleles, and the tools for mutation identification by WGS are tailored to that end.

We reasoned that the data produced by WGS, coupled with the appropriate mapping crosses, should greatly enhance our ability to identify causative variants among the less genetically tractable categories of mutations. To that end, we have utilized a polymorphism mapping method previously developed for mutation identification in *C. elegans* (Wicks *et al.* 2001; Swan *et al.* 2002; Doitsidou *et al.* 2010), and evaluated various strategies for mapping dominant, semidominant, and two-gene mutations. We have also adopted a method to prepare sequencing libraries from small amounts of genomic DNA, and demonstrate its utility in strains where sample recovery may be limiting. Finally, we have performed analysis of WGS data from traditional recombinant mapping crosses, useful for mutation identification in legacy strains or in cases where polymorphism mapping may not be suitable. Together, those approaches extend the application of WGS to the identification of various types of mutations.

## Materials and Methods

All *C. elegans* mutant strains were derived from the wild-type N2 Bristol strain and contained one or more of the following alleles: *rol-6(su1006) II*, *lin-8(n111) II*, *lin-9(n112) III*, *spe-48(hc85) I* (formerly identified as *spe-8*), *spe-10(hc104) V*, *dpy-11(e224) V*, *unc-76(e911) V*. Hawaiian strain isolate CB4856 (Hodgkin and Doniach 1997) was used for polymorphism mapping. CRISPR-mediated gene editing to create *spe-48(gd11)* was performed as described previously (Paix *et al.* 2015), using *dpy-10* as a co-CRISPR marker (Arribere *et al.* 2014). CRISPR reagents are listed in Supplemental Material, Table S1. Worms were propagated using standard growth conditions (Brenner 1974). Fertility assays were performed as described previously (Kulkarni *et al.* 2012).

**Figure 1:**
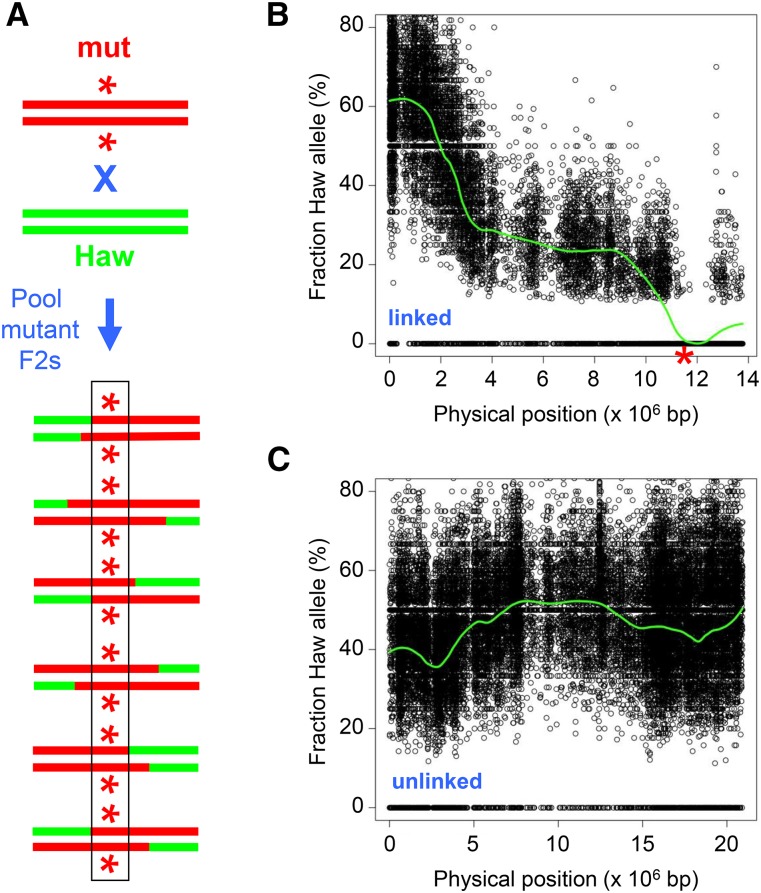
Polymorphism mapping of recessive mutations. A. Mapping strategy. Only the mutation-bearing chromosome is diagrammed. The strain containing the mutation of interest (asterisk, in red) is crossed to the polymorphic mapping strain (Haw, in green). F2 progeny homozygous for the mutation are pooled for sequencing. B. Mapping data. The proportion of reads corresponding to each Hawaiian SNP is plotted against the physical map. The green line is a LOESS regression of the SNP frequency. Shown is a representative plot used to map the APC subunit mutation *such-1(ax2010)* at chromosome III, position 11.5 Mb (indicated by red asterisk) (Wang *et al.* 2014). C. Mapping data from the same strain of an unlinked chromosome (chrV), for comparison.

Sequencing libraries were constructed using the TruSeq DNA sample prep kit v2 for large-input gDNA samples or TruSeq ChIP sample prep kit for low-input gDNA samples (Illumina, San Diego, CA). Briefly, gDNA samples were sheared by sonication, end-repaired, A-tailed, adapter-ligated, and PCR-amplified. A detailed protocol for obtaining sheared gDNA from small numbers of animals can be found in File S1. Libraries were sequenced on a HiSeq 2500 (Illumina, San Diego, CA) to generate single-end 50-bp reads, and yielded >20-fold genome coverage per sample. The reference genome used for alignment was obtained from WormBase version WS220 (www.wormbase.org). For Hawaiian single-nucleotide polymorphism (SNP) mapping, we used a list of 112,061 annotated Hawaiian SNPs (Wicks *et al.* 2001; Swan *et al.* 2002; D. Spencer and R. H. Waterston, unpublished data) from WormBase version WS220. The SNP data in Figure 1 were determined with a pipeline of BFAST (Homer *et al.* 2009) for alignment and SAMtools (Li *et al.* 2009) for variant calling. All other SNP data were obtained using BBMap (Bushnell 2015) for alignment and FreeBayes (Garrison and Marth 2012) for variant calling. Duplicate reads were removed after alignment and, unless otherwise indicated, at least three independent reads were required for a variant call. ANNOVAR (Wang *et al.* 2010) was used for annotation. For polymorphism mapping, SNP frequencies with LOESS regressions were plotted against chromosome position with R (R Core Team 2015) using the same parameters reported for CloudMap (Minevich *et al.* 2012).

### Data availability

Sequence data are available at the NCBI Sequence Read Archive (BioProject accession number PRJNA305991).

## Results and Discussion

### SNP mapping strategy

We employed a previously described one-step method for simultaneously mapping and identifying candidate mutations in *C. elegans* by WGS (Doitsidou *et al.* 2010). The parental strain bearing the mutation of interest is mated to a highly polymorphic Hawaiian strain isolate, which contains ∼370,000 SNPs (Thompson *et al.* 2015). For mapping, we used a subset of ∼110,000 (roughly one per kbp) previously annotated SNPs (see *Materials and Methods* for details). Recombination between the parental and Hawaiian chromosomes occurs in the heterozygous F1 hermaphrodites, which are allowed to self-fertilize. Those F2 progeny that exhibit the mutant phenotype (mutant F2s, for short) are selected and pooled for sequencing. The positions and frequencies of the Hawaiian SNPs are plotted on the physical map; the SNPs are present in ∼50% of the reads at unlinked loci, but are absent in the interval where the mutation resides (Figure 1, A−C). The same data are used to identify novel homozygous sequence variants within the mapping interval as candidate mutations. The strategy has been used successfully to identify a number of recessive mutations (*e.g.*, Doitsidou *et al.* 2010; Labed *et al.* 2012; Liau *et al.* 2013; Connolly *et al.* 2014; Wang *et al.* 2014; Jaramillo-Lambert *et al.* 2015). With the exception of the strains that contain linked morphological markers (see below), we utilized the Hawaiian SNP mapping method described here for the characterization of different categories of mutations.

### Dominant and semidominant alleles

For recessive alleles, homozygosity in the mutant F2s produces a gap in the Hawaiian SNPs that defines the mapping interval. Dominant alleles, however, yield a mixture of heterozygous and homozygous mutant F2s in a 2:1 ratio. Consequently, the frequency of Hawaiian SNPs at the mutant locus is only slightly reduced, from 50 to 33%, with a concomitantly small increase in the candidate allele frequency to 67%. Therefore, neither the mapping interval nor the causative mutation is clearly defined.

**Figure 2:**
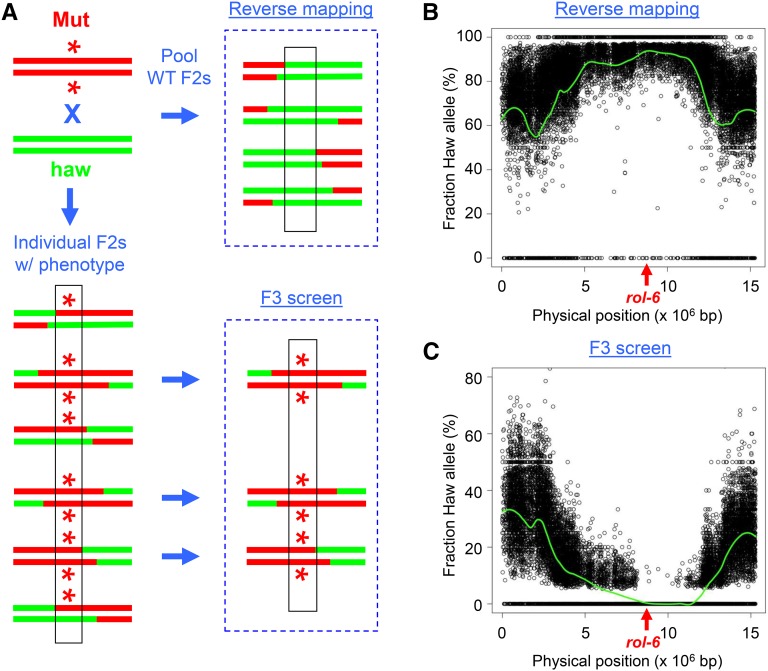
Polymorphism mapping of dominant mutations. A. Mapping strategies. The strain bearing the dominant mutation (asterisk, in red) is crossed to the mapping strain (haw, in green). For reverse mapping, wild-type F2 progeny are pooled for sequencing. For F3 screening, progeny from individual mutant F2s are screened to identify homozygous mutants, which are pooled for sequencing. B. Reverse mapping data for *rol-6(su1006)*. Plot is shown for chromosome II, which contains *rol-6* at position 8.7 Mb. C. Mapping data for F3 screen. Chromosome II, with *rol-6* indicated.

We considered two different strategies to address the problem (diagrammed in Figure 2A). For the first strategy, reverse mapping, wild-type F2s are picked and pooled for sequencing. The dominant allele is excluded from the population, so the Hawaiian SNP frequency rises to 100% in the mapping interval. An additional sequencing sample, from the mutation-bearing parental strain, is required to identify candidate mutations in that region. The second strategy involves the screening of F3 progeny from individual mutant F2s, to discriminate homozygous (100% mutant F3s) from heterozygous (75% mutant F3s) F2 animals. The homozygotes can then be pooled, sequenced, and analyzed in the same manner as recessive mutations. F3 screening has been suggested previously (Doitsidou *et al.* 2010) but unsupported by data, so we chose to evaluate its performance relative to reverse mapping.

We tested the two mapping strategies using the well-characterized *rol-6(su1006)* mutation. The *rol-6* gene encodes a cuticular collagen, and the *su1006* allele produces an easily identifiable dominant roller (Rol) phenotype (Kramer *et al.* 1990). For the reverse mapping strategy, we picked 100 wild-type F2 progeny from the mapping cross. The mapping plot produced an elevated frequency of SNPs on a single chromosome (chrII) with a peak that encompasses *rol-6* (Figure 2B). For the F3 screen, we picked 120 individual F2 rollers and identified 36 homozygotes that produced 100% Rol progeny. Polymorphism mapping revealed a reduction in SNP frequency on chromosome II, with a gap that encompasses *rol-6* (Figure 2C). Our results demonstrate the validity of the two methods for mapping dominant mutations.

We note that the mapping interval from reverse mapping is not as clearly demarcated as for F3 screening, because the latter approach benefits from a threshold effect for SNP detection. First, low-frequency SNP detection is limited by read depth; for 20-fold genome coverage, the average limit is 5% (1/20). Second, the presence of sequencing errors in the data produces a large number of low-frequency false-positive SNP calls. Filtering by frequency removes those false positives, but also removes *bona fide* low-frequency SNPs. Consequently, a discontinuity is produced in the SNP plot (in Figure 2C, note the absence of SNPs at the bottom of the plot between 0% and ∼5%) and creates a gap that defines the mapping interval. By contrast, the reverse mapping method is unaffected by low-frequency SNPs, and produces a continuous plot that approaches the maximum frequency (Figure 2B, top). To better visualize the peak for reverse mapping, we calculated the normalized frequency of homozygous SNPs in 0.5 Mb intervals (Figure S1; see legend for details). A histogram of those values indicated that the peak SNP frequency corresponds with the location of *rol-6*. We conclude that reverse mapping is at least as accurate as F3 screening for dominant alleles.

The two methods that we describe for dominant mapping provide different advantages for the user. F3 screening is more cost-effective; only a single sequencing sample is needed for both mapping and mutation identification, whereas reverse mapping demands two sequencing samples. However, F3 screening requires that the mutant phenotype be observable in two sequential generations, and many classes of mutations do not fulfill that criterion. Phenotypic categories unsuitable for F3 screening include embryonic or larval lethality or arrest, sexual transformation leading to infertility, gametogenesis defects, and male-specific phenotypes. Dominant mutations have been reported for several of those classes: *mel-26(ct61)* and *mei-1(ct46)* result in maternal-effect embryonic lethality (Mains *et al.* 1990), 21 alleles of *tra-1(gf)* produce sterile females (Hodgkin 1987), and six alleles of *wee-1.3* cause sperm-specific sterility (Lamitina and L’Hernault 2002). Mutations such as those are refractory to mapping by F3 screening but accessible by reverse mapping.

In principle, semidominant alleles should be more amenable to analysis than dominant alleles, since heterozygous animals exhibit an intermediate phenotype that can be distinguished from homozygotes. In practice, many phenotypes (such as body length or lifespan) are phenomena with a continuous range of values, and it may not be possible to assign the genotype of individuals unambiguously based on the observed characteristic. Therefore, we recommend the same F3 progeny screening strategy used for dominant alleles, which should prove similarly sufficient to distinguish the zygosity of individual F2 animals. The reverse mapping strategy might also be employed, but only in cases where the wild-type and heterozygous phenotypes are fully resolved.

### Two-gene synthetic interactions

The category is defined by the requirement for mutations at two loci to produce the phenotype of interest. Although more typically isolated as genetic enhancers, it is also possible to obtain such double mutants by unbiased forward screening. One of the earliest screens in *C. elegans* for cell lineage mutants recovered the recessive *lin-8(n111)*; *lin-9(n112)* pair of alleles that define the synthetic multivulva (synMuv) class A and B categories (Ferguson and Horvitz 1989). Each of those mutations is phenotypically silent in isolation, but the double mutant exhibits multiple ectopic pseudovulvae (Muv phenotype).

**Figure 3:**
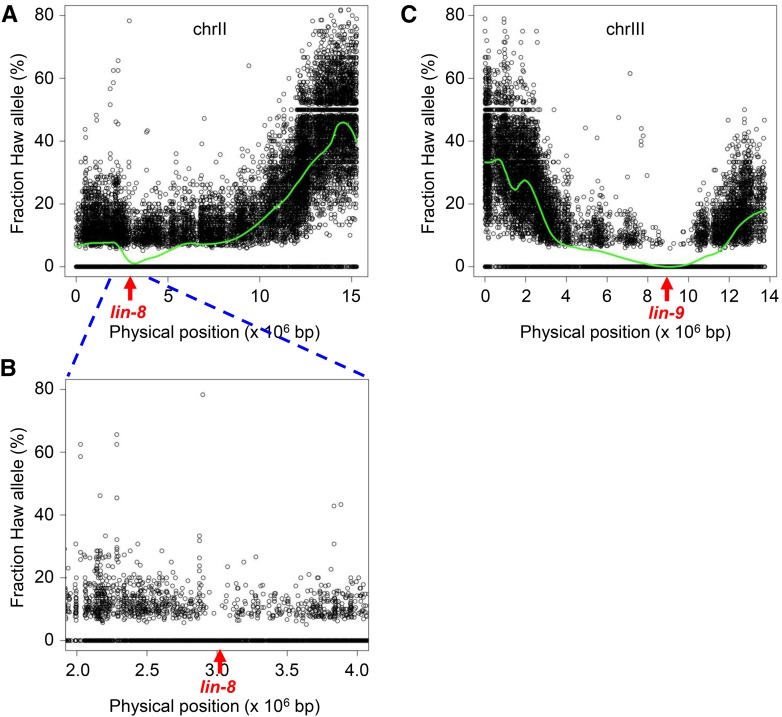
Polymorphism mapping of *lin-8*; *lin-9* double mutations. SNP mapping plots. A. Chromosome II, which contains *lin-8(n111)* at position 3.1 Mb. B. Enlargement of the chromosome II interval from 2.0 to 4.0 Mb. C. Chromosome III, which contains *lin-9(n112)* at position 8.9 Mb.

We chose the *lin-8*; *lin-9* pair as a test case for the application of WGS to the identification of two-gene synthetic interactors. We selected 100 F2 Muv progeny from the mapping cross and pooled them for sequencing. Two chromosomes contained a biased distribution of SNPs, with mapping intervals that correspond to the positions of *lin-8* and *lin-9* (Figure 3, A−C). The gap in SNPs flanking *lin*-8 is masked by a high local density of polymorphisms, but evident at higher resolution (Figure 3B). We note that the same method has been used to map the double mutation *ot628*, obtained in a screen for loss of neuronal identity (O. Hobert, personal communication). We conclude that WGS and polymorphism mapping can be applied successfully to map two-gene traits.

### Sequencing from small samples

Many categories of mutations produce terminal phenotypes (such as lethality or sterility) that effectively limit the sample size. Standard WGS library preparation calls for microgram quantities of input gDNA, which can only be obtained from large populations. However, protocols have been developed for library construction that require only low nanogram amounts of DNA, so we evaluated the suitability of low-input libraries for mutation identification from small numbers of worms.

A major concern is that the sequence coverage obtained by using low-input library preparation might prove insufficient for our application. Accurate mutation identification requires data that encompass the entire genome. The potential for coverage bias from small amounts of input DNA might preclude detection of some variants. To address that concern, we compared the data produced from a standard library preparation using 1 µg of gDNA (referred to as the large sample) to those generated from a low-input library prep protocol using only 2 ng (hereafter, small sample) of the same gDNA. We selected the highly polymorphic Hawaiian strain as our test case, to ensure a large number of validated SNPs for identification. We used equal numbers of sequence reads for the two data sets and compared the alignment metrics.

**Figure 4:**
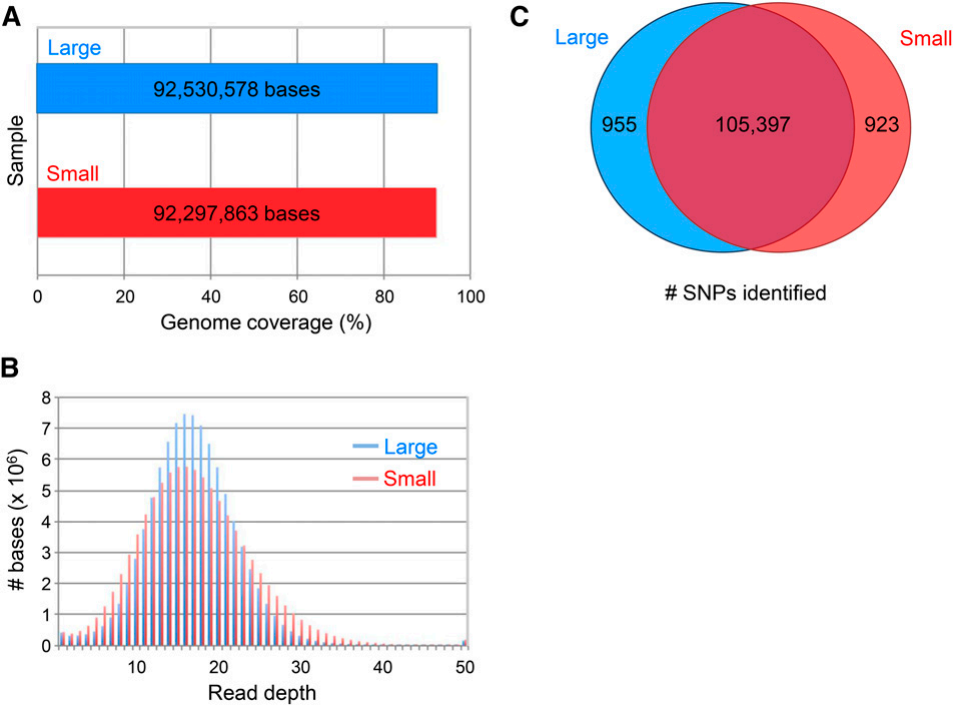
Coverage for large *vs.* small-input Hawaiian samples. A. Number of bases in the reference genome covered by at least three uniquely mapped reads. B. Read depth distribution for the large (blue) and small (red) input samples. C. Venn diagram indicating the number of Hawaiian SNPs identified in each sample.

First, we determined the fraction of the genome that was covered by at least three reads (the minimum read depth required for a variant call in our analysis) in the large and small samples. We observed little difference (<0.25%) in genome coverage between the two libraries (Figure 4A), indicating that the small sample does not introduce substantial gaps in coverage. Note that our coverage calculations are conservative; repetitive sequences constitute ∼5% of the genome and, by filtering reads that mapped to multiple loci, those sequences were excluded. Next, we considered more subtle evidence for biased coverage. The small amount of input DNA might reduce the complexity of that library relative to the large sample, producing an increase in the fraction of duplicate reads as well as a larger variance in the per-base depth of coverage. We did observe differences in the degree of read duplication (large, 15.8%; small, 39.1%) as well as the depth of coverage distribution between the two libraries (Figure 4B), consistent with reduced library complexity in the small sample. However, the impact of those differences on SNP detection was minimal. After removing the duplicate reads from each sample, we performed variant calling and compared the lists of annotated Hawaiian SNPs that were identified in each data set. The numbers and identities of the SNP calls were essentially the same between the large and small samples, with greater than 99% overlap (Figure 4C). We conclude that libraries constructed from small amounts of input are sufficient for accurate mutation identification.

**Figure 5:**
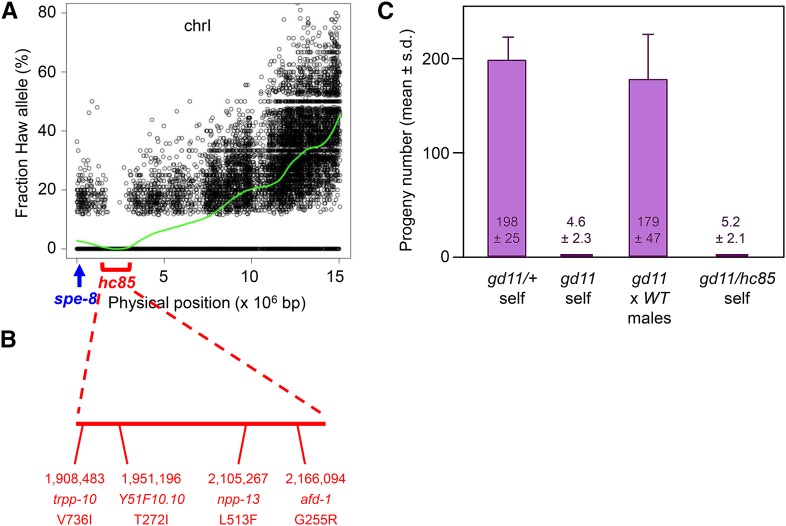
Polymorphism mapping from small input sample. A. Mapping data for *spe-48(hc85)* allele. Mapping interval (red bracket highlighting SNP gap at 1.7−2.7 Mb) differs from position of *spe-8* (blue arrow at position 0.1 Mb). B. Physical map of candidate mutations in the *spe-48(hc85)* mapping interval. Position, gene name, and amino acid change are indicated. C. Fertility data from the CRISPR allele *spe-48(gd11)*. Mean progeny counts with standard deviations (n = 10 hermaphrodites) are shown for the indicated genotypes.

We proceeded to obtain gDNA from a small population of worms with a terminal mutant phenotype. Our test strain contained the recessive *hc85* allele, which produces a sperm-specific defect that renders them sterile (L’Hernault *et al.* 1988). The mutation was originally assigned to the *spe-8* complementation group. However, more recent data dispute that assignment; *hc85* complements the sperm-specific sterility of another *spe-8* allele, and targeted sequencing of the *spe-8* gene from the strain bearing *hc85* failed to identify a molecular lesion (Muhlrad *et al.* 2014). To address that discrepancy, we used the Hawaiian mapping cross and recovered gDNA from a pool of 50 hand-picked sterile F2 animals. SNP mapping defined the interval between positions 1.7 and 2.7 Mb on chromosome I (Figure 5A). That interval is distinct from the position of *spe-8*, which is located on the same chromosome at position 0.1 Mb.

We confirmed the validity of the SNP mapping data by determining the molecular identity of the *hc85* allele. Four genes within the mapping interval contained novel, homozygous, nonsynonymous mutations (Figure 5B). Previous analyses determined that virtually all Spe genes exhibit sperm-specific expression (*e.g.*, see Kulkarni *et al.* 2012), so the four candidates were prioritized by that criterion. Recently published RNA-Seq data (Ma *et al.* 2014) indicated that only one of those four genes, *Y51F10.10*, is detectably expressed in sperm. Therefore, we engineered the observed *Y51F10.10* missense mutation, a threonine-to-isoleucine substitution at amino acid 272, into the wild-type strain via CRISPR-mediated gene editing (Paix *et al.* 2015). The engineered allele, designated *gd11*, recapitulates the recessive Spe phenotype of *hc85*. Self-fertility is normal for heterozygous *gd11/+* hermaphrodites but low for homozygous *gd11* hermaphrodites, and fertility is restored by mating to wild-type males (Figure 5C). Complementation testing of the Spe phenotype revealed the *gd11* and *hc85* mutations to be allelic, as self-fertility of *gd11/hc85* hermaphrodites is low (Figure 5C). Because the *Y51F10.10* gene is distinct from *spe-8*, we have assigned the new gene name *spe-48* to *hc85* and *gd11*.

### Marked mutant mapping

The examples described above require mating to the polymorphic Hawaiian strain to identify the mapping interval. However, there are numerous cases where that cross may not be suitable. First, there are known genetic incompatibilities between the Hawaiian strain and the standard laboratory N2 Bristol strain (Seidel *et al.* 2008). The incompatibility loci produce biased segregation ratios in the F2 population, such that mutations in the vicinity of the loci are difficult to map (Minevich *et al.* 2012). Second, the genetic diversity of the polymorphic strain might include modifiers of the phenotype of interest, thereby confounding the isolation of phenotypically identifiable animals needed to generate the mapping information. Finally, the investigator may wish to take advantage of available recombinant lines obtained from crosses to morphologically marked strains. Those include legacy strains that were mapped using two- or three-factor crosses, as well as lines constructed to link an easily visible marker to the mutation of interest.

**Figure 6:**
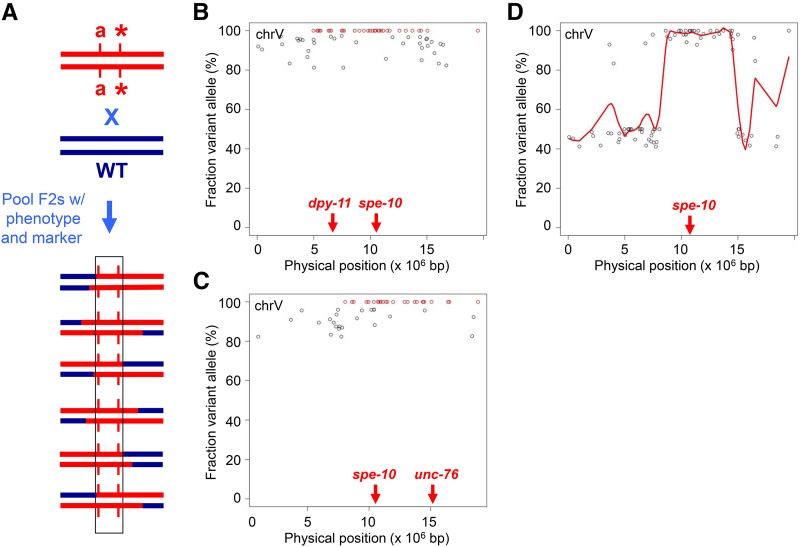
Mapping of mutations linked to markers. A. Mapping strategy. The strain bearing the marker mutation (*a*, in red) and linked mutation of interest (asterisk) is crossed to wild-type (blue). F2s homozygous for both mutations are pooled for sequencing. B−C. Mapping data for *dpy-11 spe-10* and *spe-10 unc-76*, respectively. Homozygous variants (>80% variant call) unique to the mutant strain are plotted against the physical map. Red points, 100% variant calls. D. Reciprocal mapping. Data from B and C are combined, and average variant fraction is plotted against the physical map. Red line, LOESS regression of the average variant fraction.

A map-free method of mutation identification has been developed (Zuryn *et al.* 2010), in which homozygosity of linked variants after extensive backcrossing defines the mutation interval. An extension of that approach, using bulk segregant analysis, was subsequently reported (Abe *et al.* 2012; Minevich *et al.* 2012). We propose a conceptually similar strategy for the analysis of mutations linked to a morphological marker (Figure 6A). Those strains contain two sources of novel genetic variation that are absent from the wild-type strain: (1) *de novo* variants produced during mutagenesis, and (2) pre-existing variants in the morphologically marked strain. A single backcross of the marked mutant strain to the wild-type is used to determine the mapping interval. Nonrecombinant F2 segregants are homozygous for both marker and mutation, and any variants between those loci. Sequencing of the nonrecombinant F2s and comparison to the wild-type strain sequence is used to discriminate common *vs.* novel homozygous variants; a plot of the latter reveals a cluster that defines the mapping interval. Note that the strategy is not predicated on knowledge of the physical location or molecular identity of the linked morphological marker allele, nor does it require accurate determination of the recombination frequency between the marker and the mutation of interest.

We evaluated the strategy using the *spe-10(hc104)* mutation marked with *dpy-11(e224)*. The former confers temperature-sensitive, sperm-specific sterility (Spe phenotype), and the latter produces short, fat animals (Dpy phenotype). We crossed the *dpy-11spe-10* strain to wild-type males, and picked 50 Spe Dpy F2s grown at the restrictive temperature for sequencing. After variant calling, we removed those that were also present in the wild-type sample and identified novel homozygous variants (minimum 15 reads, >80% variant call). The 80% threshold accommodates errors in the sequence data as well as mistakes in selecting the desired F2s, at the expense of including some nonhomozygous variants. The distribution of homozygous variants was clearly enriched on chromosome V (73 of 87 total, or 84%). We plotted those variants and highlighted the *bona fide* (100%) homozygous calls (Figure 6B, in red), and observed a cluster between 5.0 and 14.5 Mb (a span that encompasses *dpy-11* at 6.5 Mb and *spe-10* at 10.4 Mb). We conclude that, in the case of strains bearing marked mutations, a single backcross is sufficient for mapping.

Use of a marked mutation offers some advantages over the previously described non-Hawaiian mapping strategies. It employs only a single round of backcrossing, which is less laborious than the multiple rounds of backcrossing necessitated by the initial map-free method of Zuryn *et al.* (2010). It also requires less effort than bulk segregation analysis: only the marked segregants are screened for the mutant phenotype, which reduces the number to be screened by ∼75%. That feature is particularly useful for mutations that are difficult to score or exhibit low/variable penetrance. Our strategy also foregoes the additional work and expense of sequencing the background strain that is used for filtering in bulk segregation analysis (Minevich *et al.* 2012), albeit with the disadvantage of retaining background mutations that are not causative. However, our method was designed specifically with legacy mutations in mind, to accommodate cases where background filtering may not be suitable (*e.g.*, the provenance of the marked mutant strain is uncertain, the background strain is no longer available, or the mutant strain has drifted significantly since its original isolation). In instances where multiple strains are analyzed from the same mutant screen, it would still be possible to filter background variants that are present in more than one strain.

A disadvantage of marked mutant mapping is that the mapping interval must span both loci, which can produce an unacceptably large mapping interval if the marker and mutation are not tightly linked. In some cases, it might be preferable to use only recombinant F2 segregants to provide higher-resolution mapping. However, screening for F2 recombinants may entail substantially more work. In the example provided, the desired recombinant (*dpy-11spe-10/+spe*-10) is morphologically wild-type but sterile, and the map distance between the two loci is 2.54 cM. To obtain the 50 animals needed for small-input library construction as above, it would require screening of ∼4000 (50/0.5 × 0.0254) wild-type F2s. Thus, the tradeoff between mapping resolution and additional screening will be dictated by the particulars of the mutant phenotype.

Higher-resolution mapping can also be obtained in cases where reciprocal recombinants are available from three-factor crosses with flanking markers. Individual homozygous mutant lines containing either the left or right marker are sequenced separately, and the frequencies of novel variants in each sample are plotted on the physical map as above. A contiguous cluster of homozygous variants present in both samples defines the mutation interval. The endpoints are delimited by variants that are homozygous in only one sample.

We assessed the utility of reciprocal mapping with a *spe-10(hc104) unc-76(e911)* strain (the latter mutation produces uncoordinated, or Unc, movement). After mating with wild-type males, we picked 50 Spe Unc F2s grown at 25° for sequencing. First, we mapped homozygous variants as above. The plot revealed a biased distribution on chromosome V (52 of 69 total variants, 75%), with a cluster between 8.0 and 16.5 Mb that contains *spe-10* and *unc-76* (located at 15.1 Mb; Figure 6C). For reciprocal mapping, we used the homozygous variants identified in either the *dpy-11spe-10* sample or the *spe-10unc-76* sample, and calculated the average variant fraction by combining the data from both samples. The map plot produced a smaller interval than either single sample, with well-defined endpoints at 8.6 and 14.5 Mb clearly indicated by the LOESS curve (Figure 6D). The utility of the strategy should be weighed against the effort and expense of sequencing an additional strain, but it may be worthwhile when the markers are far from the mutation of interest and/or validation of candidate alleles is challenging.

### Conclusions

WGS has transformed our ability to identify mutations obtained from forward screening. We describe a variety of methods for applying the technology to types of mutations – dominant and semidominant alleles, synthetic interactors, and terminal phenotypes – that pose particular challenges to identification. We demonstrate the validity of those methods by the identification of both previously known as well as new mutations, and confirm the latter by independent criteria. Although our analyses were limited to *C. elegans*, the strategies can be generalized to any species in which polymorphic isolates are available for crosses and should prove useful for researchers who wish to apply the power of WGS to the investigation of alleles that are difficult to identify by other methods.

## Supplementary Material

Supplemental Material
