# Supplementary material for: Mapping Challenging Mutations by Whole-Genome Sequencing
Source: G3 (Bethesda). 2016 Mar 4;6(5):1297–304. doi: 10.1534/g3.116.028316 (PMC4856081; doi:10.1534/g3.116.028316)
Supplement: Supplemental Material [file supp_6_5_1297__index.html]

Mapping Challenging Mutations by Whole-Genome Sequencing — Supplemental Material 

# Mapping Challenging Mutations by Whole-Genome Sequencing

## Supplemental Material for Smith *et al.*, 2016

**Files in this Data Supplement:**

- File S1 - Small-scale worm DNA prep for sequencing library. (.pdf, 79 KB)
- Figure S1 - Reverse mapping of dominant mutations. (.pdf, 361 KB)
- Table S1 - Reagents for CRISPR-mediated gene editing. (.pdf, 313 KB)
