# Supplementary material for: Mapping Challenging Mutations by Whole-Genome Sequencing
Source: G3 (Bethesda). 2016 Mar 4;6(5):1297–304. doi: 10.1534/g3.116.028316 (PMC4856081; doi:10.1534/g3.116.028316)
Supplement: Supplemental Material [file supp_g3.116.028316_FileS1.pdf]

File S1. Small-scale worm DNA prep for sequencing library.

1. Pick worms (at least 50 adults) into a 1.5mL centrifuge tube containing 500 µL M9.

2. Vortex briefly (3-5 seconds), then spin 60 seconds @ 1300 RCF.

3. Remove most of the M9 by pipette, taking care to avoid the worms.

4. Resuspend in 500 µL M9; repeat the wash at least three times to remove bacteria.

5. Resuspend in 500 µL M9; incubate 30 minutes with gentle agitation to allow digestion/elimination of bacteria from the intestine; spin and remove most of the M9.

6. Perform a final wash with 500 µL TE; spin one minute at top speed; remove TE, leaving ~100 µL.

7. Add 400 µL worm lysis buffer to the worm sample; mix briefly.

8. Sonicate with a BioRuptor (Diagenode) using the following settings: high power; 30 seconds on/30 seconds off; 2 x 15 minutes sonication time.

9. Add 50 µL 10 mg/mL proteinase K; mix well; incubate one hour @ 65˚C, vortexing briefly at 10-15 minute intervals to maintain suspension.

10. Add 20 µL 10 mg/mL RNase A; mix well; incubate 30 minutes @ 37˚C.

11. Purify sheared gDNA using a MinElute column (Qiagen) per the manufacturer's protocol; the final elution volume is 10 µL.

Recipes

<u>M9 buffer</u>
3 g KH_2PO_4,
6 g Na_2HPO_4
5 g NaCl
1 mL 1 <u>M</u> $MgSO_4$
999 mL H_2O

<u>TE buffer</u>
10 mL 1 <u>M</u> Tris-HCl, pH 8.0
2 mL 0.5 <u>M</u> EDTA, pH 8.0
988 mL H_2O

<u>Worm lysis buffer</u>
100 mL 1 <u>M</u> Tris-HCl, pH 8.0
20 mL 5 <u>M</u> NaCl
100 mL 0.5 <u>M</u> EDTA
125 mL 10% (w/v) SDS
655 mL H_2O
