# Supplementary material for: Mapping Challenging Mutations by Whole-Genome Sequencing
Source: G3 (Bethesda). 2016 Mar 4;6(5):1297–304. doi: 10.1534/g3.116.028316 (PMC4856081; doi:10.1534/g3.116.028316)
Supplement: Supplemental Material [file supp_g3.116.028316_FigureS1.pdf]

## Reverse mapping

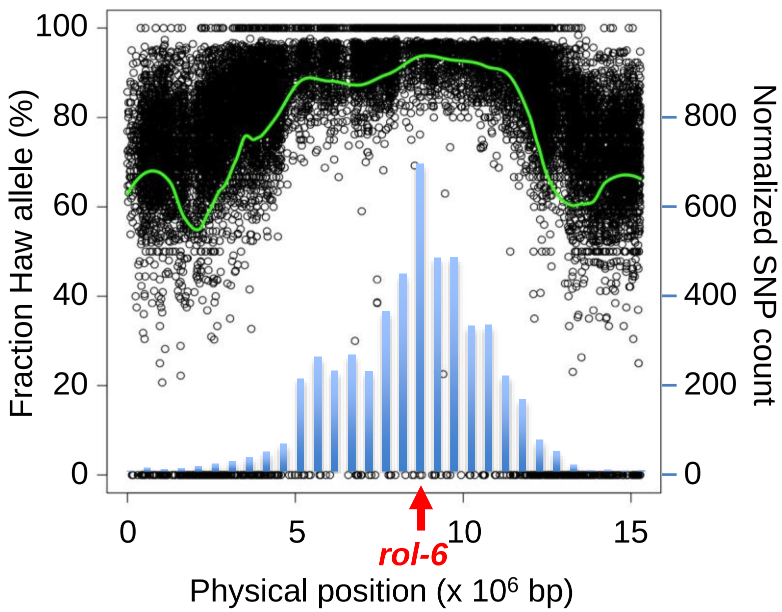


**Figure S1. Reverse mapping of dominant mutations.** Shown is the identical plot for Figure 2B, overlaid with a histogram (blue bars) of the normalized homozygous SNP count. The data were calculated in 0.5 Mb bins in the same manner as for CloudMap (Minevich *et al.,* 2012): [number of homozygous SNPs / (number of total SNPs – number of homozygous SNPs)] x average number of total SNPs per bin, per chromosome.
