# Supplementary material for: Mapping Challenging Mutations by Whole-Genome Sequencing
Source: G3 (Bethesda). 2016 Mar 4;6(5):1297–304. doi: 10.1534/g3.116.028316 (PMC4856081; doi:10.1534/g3.116.028316)
Supplement: Supplemental Material [file supp_g3.116.028316_TableS1.pdf]

**Table S1. Reagents for CRISPR-mediated gene editing.**		
**Reagent**	**Sequence (5’-3’)**	**Concentration injected**
Y51F10.10 crRNA	UUCAUCAAUGGAGCUUUUCCGUUUUAGAGCUAUG CUGUUUUG	8 μg/mL
Y51F10.10 repair DNA	ATTTTTACAGATTGAATACTACATGAAAGCATGCC GAACAGTCGAAAAAATCGGGAAGAGCTCCATTGAT GAAACGTTGCCGTTCTTGATTCAATGCAATT[1]	0.6 μg/mL
dpy-10 crRNA	GCUACCAUAGGCACCACGAGGUUUUAGAGCUAUG CUGUUUUG	8 μg/mL
dpy-10 repair DNA	CACTTGAACTTCAATACGGCAAGATGAGAATGACT GGAAACCGTACCGCATGCGGTGCCTATGGTAGCGGA GCTT CACATGGCTTCAGACCAACAGCCTAT	0.6 μg/mL
tracrRNA	AACAGCAUAGCAAGUUAAAAUAAGGCUAGUCCGU UAUCAACUUGAAAAAGUGGCACCGAGUCGGUGCU UUUUUU	4 μg/mL

[1]Edits introduced into *Y51F10.10* by CRISPR are underlined and indicated in red; ‘T’ replaces ‘C’ and introduces the *hc85* mutation (Thr272Ile); ‘G’ replaces ‘A’ as a synonymous mutation that introduces a *Sac*I restriction site for screening.
